# Patients with infectious TB who resist and refuse isolation in wards in Japan, 2022–2024

**DOI:** 10.5588/pha.25.0022

**Published:** 2025-12-03

**Authors:** Y. Nagata, M. ota, T. Zama, S. Hirao

**Affiliations:** Research Institute of Tuberculosis, Japan Anti-Tuberculosis Association, Tokyo, Japan.

**Keywords:** tuberculosis, Japan, epidemiology, infection control

## Abstract

**BACKGROUND:**

In Japan, a low-TB-burden country, approximately 4000 cases of sputum smear-positive TB are reported annually and the patients are typically isolated in a TB ward until they become smear-negative. However, there are some patients who resist or refuse isolation. This study aims to characterize these patients.

**METHODS:**

A descriptive study. A self-administered questionnaire was sent to local health offices about patients registered from April 2022 to March 2024 who resisted or refused isolation.

**RESULTS:**

A total of 71 patients (0.99%) who resisted or refused isolation were identified among 7,186 with smear-positive TB in the study period. In 2022, 22 (31.0%) such cases were reported, whereas there were 49 (69.0%) in 2023. Fifty-seven of these patients (80.3%) were male, with age that peaked in their 70s, 61 (85.9%) were born in Japan, and 28 (39.4%) were unemployed. Tokyo, the capital, reported 13 (18.3%) such cases, followed by Osaka (12, 16.9%) and Saitama (8, 11.3%) prefectures, whereas 24 (51.1%) of 47 prefectures reported none.

**CONCLUSION:**

Although the number of patients with TB who resisted or refused isolation was small, there should be one or two TB facilities with law enforcement officials readily available to enforce isolation.

In Japan, the TB notification rate has declined 85-fold over the past seventy years, from 698/100,000 population in 1951 to 8.2/100,000 in 2022.^1^ Nevertheless, approximately 4,000 sputum smear-positive TB cases are still reported annually, with over 65% of these patients being older than 65 years.^1^ This indicates a shift in the infection pool from younger or middle-aged individuals to the elderly population.^2,3^ Another concern related to TB control is TB among immigrants from countries with a high-burden of TB. Outbreaks involving the elderly and immigrants have been reported.^4–11^ The 1998 Act on Prevention of Infectious Diseases and Medical Care for Patients with Infectious Diseases mandates the isolation of all patients with infectious (often sputum smear-positive) pulmonary TB in designated hospitals equipped with TB isolation beds.^12^ This measure aims to prevent community transmission of the disease and outbreaks.^13^ Upon a physician's notification of an individual with infectious TB to the prefectural governor, the medical director of the local health office typically issues an isolation recommendation to the patient, which is generally accepted.^13^ During the hospitalisation period, the national and local governments, alongside the health insurance agency, cover all medical expenses related to anti-TB treatment.^12^ Patients are discharged from isolation when achieving at least three consecutive negative sputum smear results. Nevertheless, a minority of patients with TB have historically exhibited resistance to, and ultimate refusal of, isolation.

In 2004, the Ministry of Health, Labour and Welfare (MHLW) of Japan conducted a survey of hospitals having TB isolation beds, inquiring about patients diagnosed with infectious TB who refused isolation. The survey revealed that 15 (0.10%) of 14,349 patients mandated for isolation throughout the year 2003 ultimately refused it.^14^ Subsequent to this, no further research has been undertaken within Japan concerning individuals with infectious TB who initially resisted isolation and/or subsequently refused adherence. The objective of this study is to delineate the characteristics of individuals with sputum smear-positive TB who, despite legal mandates for isolation in designated TB treatment facilities, exhibited non-compliance with such directives.

## METHODS

A patient with infectious TB who resisted or refused isolation was defined as a patient exhibiting sputum smear-positive TB who was directed to be hospitalised in a TB isolation bed but resisted or refused, including eventual refusal, to comply during the period from April 2022 through March 2024. In November 2024, a self-administered questionnaire was dispatched via postal mail to all local health offices throughout Japan. The list of local health offices was derived from the website of the Ministry of Health, Labour, and Welfare of Japan.^15^

The questionnaire sought to ascertain if each local health office had encountered any patients with TB who had resisted or refused isolation in a designated TB isolation bed during the study period. Affirmative responses prompted further inquiry regarding patient demographic information, the year of TB diagnosis, the rationale for resisting or refusing isolation, and subsequent follow-up procedures. Data entry was conducted twice into an Excel 2022 (Microsoft Corp, Seattle, WA, USA) spreadsheet, followed by a comparison. Discrepancies were reconciled by revisiting the original questionnaire. Fisher’s exact test was conducted to calculate the 95% confidence interval (CI) for a proportion. R software (The Foundation for Statistical Computing, Vienna, Austria) was used to calculate 95%CIs. Official surveillance data were utilized to retrieve the number of cases with sputum smear-positive TB registered from April 2022 through March 2024.^16^ Authorization to conduct the research, including ethical review, was obtained from the institutional review board of the Research Institute of Tuberculosis (#2024-09), Tokyo, Japan.

## RESULTS

We sent the questionnaire to 492 local health offices in Japan. Of these, 411 (83.5%) responded. Sixty-four health offices had experienced 71 patients with infectious TB who resisted or refused isolation during the study period. Of the 71, 54 eventually complied with the orders and were isolated, whereas 17 did not. The proportion of the patients with TB who resisted or refused isolation was 0.99% (95%CI: 0.77–1.24%) among 7186 with sputum smear-positive TB who were registered from April 2022 through March 2024 for whom isolation was considered necessary.

**FIGURE 1.**
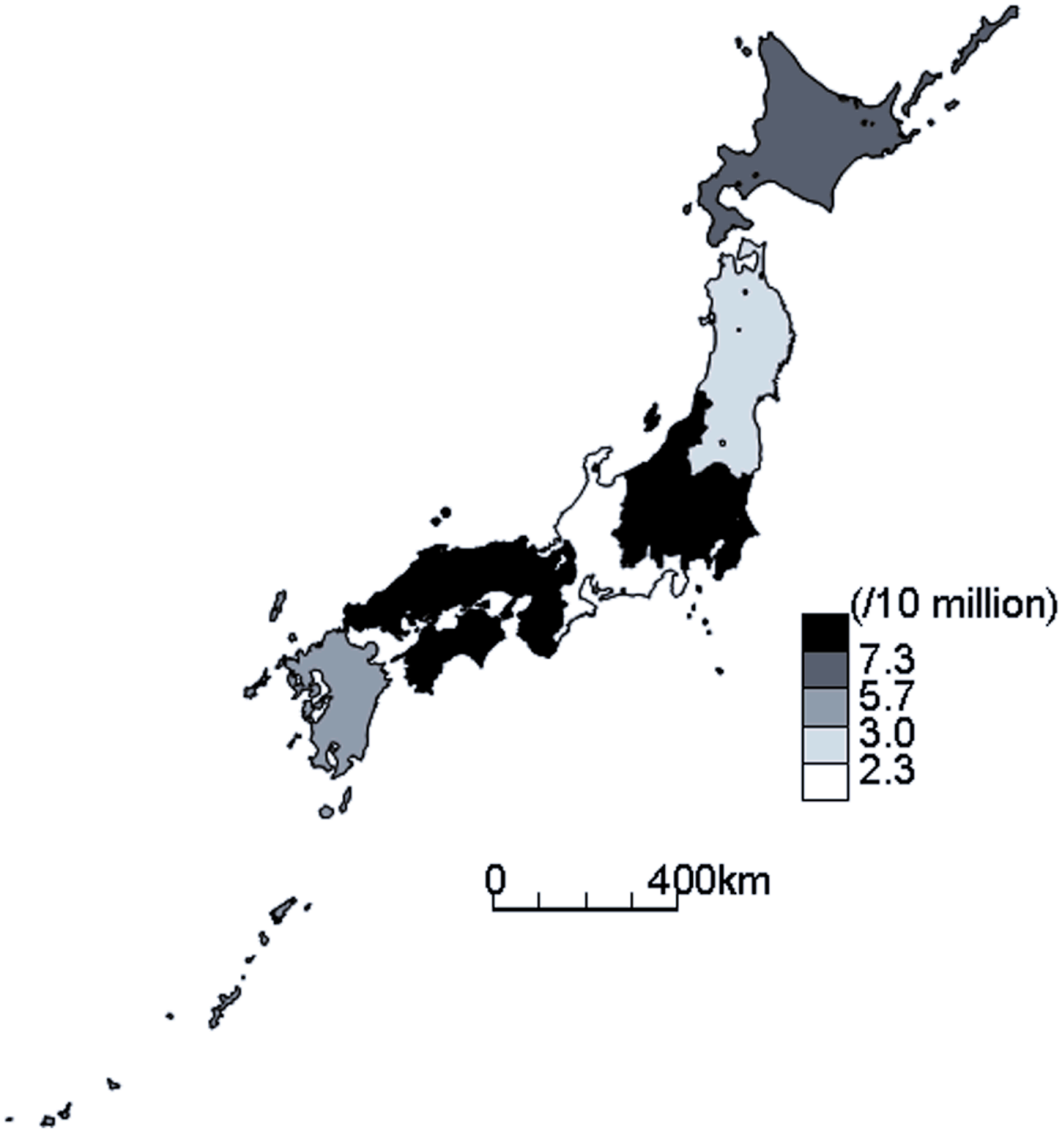
Geographic distribution of patients with infectious TB who resisted or refused isolation, Japan, 2022–2024.

**FIGURE 2.**
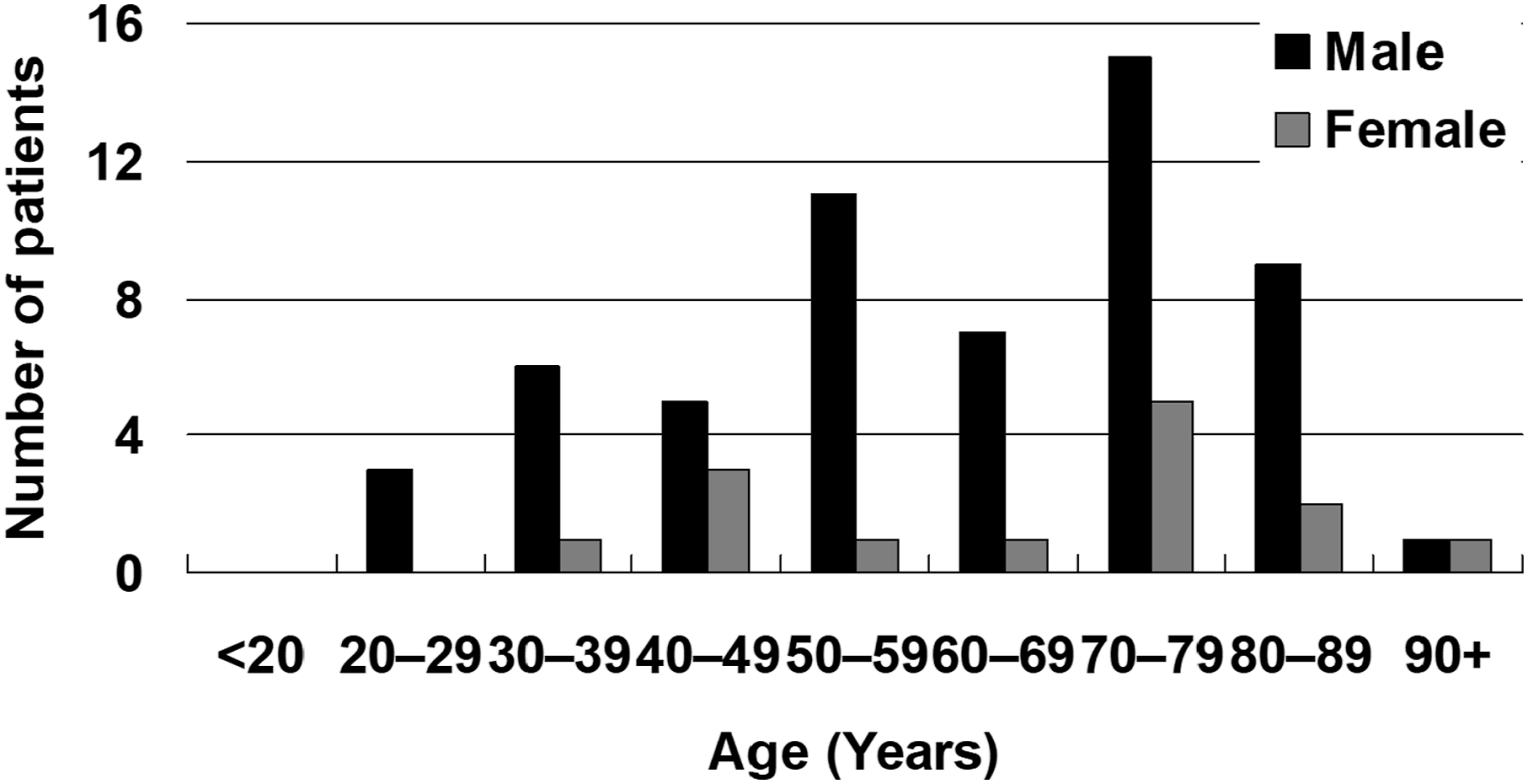
Age- and sex-distribution of patients with infectious TB who resisted or refused isolation, Japan, 2022–2024.

Tokyo, the capital, reported the highest number of patients with TB who resisted or refused isolation (13 cases, 18.3%), followed by Osaka (12 cases, 16.9%) and Saitama prefectures (8 cases, 11.3%). Conversely, 24 prefectures, representing 51.1% of the total 47 prefectures, reported no such patient. Figure 1 illustrates the geographical distribution of rates of cases with TB who resisted or refused isolation per 10 million population. The Kanto-Kou-Shin-Etsu region, encompassing the Greater Tokyo Metropolitan Area,^17^ including Saitama prefecture, exhibited the highest rate (35 cases, 8.0/10 million, 95% CI 5.6–11.1). This was followed by the Kinki region (15 cases, 7.3/10 million, 95% CI 4.1–12.0) including Osaka, and the Chugoku-Shikoku regions (8 cases, 7.3/10 million, 95% CI 3.2–14.4). On the other hand, the Tokai-Hokuriku region had the lowest rate (3 cases, 1.4/10 million, 95% CI 0.3–4.2). Compared with the Tokai-Hokuriku region, the Kanto-Kou-Shin-Etsu region had a 5.5 (95% CI 1.7–18.0) times higher rate of patients with TB who resisted or refused isolation, followed by the Kinki (5.0 times, 95% CI 1.5–17.4) and Chugoku-Shikoku (5.0 times, 95% CI 1.3–19.0) regions. Figure 2 shows the sex and age distributions of the patients with TB who resisted or refused isolation. In general, there were 4.1 times more male (n=57) than female (n=14) patients with infectious TB who resisted or refused isolation, with peaks in their 70s in both male and females. In 2022, 22 (31.0%) such patients were reported followed by 49 (69.0%) in 2023, an increase of 27 (123%). Most (61, 85.9%) such patients were born in Japan and 28 (39.4%) were unemployed. Eighteen (25.4%) had diabetes mellitus, four (5.6%) had malignant neoplasms and two (2.8%) chronic renal failure, whereas 29 (40.8%) had no complications.

Of the 71 individuals, 54 eventually adhered to the isolation directive after engagement by the local health authority personnel. Specifically, of the 54 who were isolated, 37 (68.5%) were isolated within 10 days, 10 (18.5%) between 10 and 19 days, three (5.6%) between 20 and 29 days, three (5.6%) after a period exceeding 30 days, and the time frame for one (1.9%) individual remains unknown. Initial refusal of isolation was attributed to work-related reasons by 16 individuals (31.4%), family matters by nine individuals (16.7%), financial/economic reasons by six individuals (11.8%), having no family member to spread TB to by four individuals (7.8%) and a variety of other reasons by 19 individuals (37.3%). For this question, multiple answers were accepted.

Conversely, 17 individuals ultimately refused isolation. The proportion of these patients among the 7186 with sputum smear-positive TB who were registered in the same period was 0.24% (95%CI: 0.14–0.37%). Of these 17, six (35.3%) were registered in 2022 and 11 (64.7%) in 2023. Fourteen (82.4%) were male. One individual (5.9%) each was in the 20s, 30s, 50s, and 90s, four (23.5%) were in their 40s, two (11.8%) each were in their 60s and 80s, and five (29.4%) were in their 70s. Fourteen (82.4%) were born in Japan and eight (47.1%) were unemployed. Among the 17, five (29.4%) received care at the outpatient clinics of the hospitals designated for their isolation and completed treatment, another five (29.4%) received care at outpatient clinics other than the designated hospitals, one individual (5.9%) each was treated via telemedicine and outreach medical service, two individuals (11.8%) refused anti-TB medication and eventually died, and another two (11.8%) were lost to follow-up.

## DISCUSSION

During the 2022–2023 period, 71 patients with infectious TB exhibiting resistance to or refusal of isolation were recorded. The majority of these individuals were male, aged 60 or above, and resided in major urban centers and adjacent areas. The number of cases with infectious TB who resisted or refused isolation was higher in 2023 than in 2022. This increase was likely due to the COVID-19 pandemic in 2020–2022, which resulted in limited availability of isolation beds for cases with infectious TB and less stringent implementation of isolation orders. However, by mid-2023, the COVID-19 isolation policy was abolished,^18,19^ and the isolation policy for infectious TB was more strictly enforced. The higher proportions of resistance or refusal of isolation among persons with infectious TB in large cities may be attributed to lower peer pressure to comply with government orders and a more liberal social environment compared to rural areas.^20^ The demographic of those resisting or refusing isolation, primarily males aged 60 and older, reflects the broader demographic distribution of cases with TB, which predominantly involves men and the elderly.^1^ Previous studies on recalcitrant TB patients who were experiencing difficulties during TB bed isolation and who self-discharged or were forced to discharge before sputum smears become negative have also indicated a majority of male subjects, suggesting a potential trend among males to evade restrictions.^21,22^

Compared to the previous study conducted by MHLW for 2003, in which 0.10% of patients mandated for isolation ultimately refused it, the proportion of the patients with infectious TB who refused isolation was 2.3 times (95%CI 1.1–4.5) higher in the current study.^12^ It is difficult to determine the reasons behind this as the previous study was conducted 20 years ago and the details were not published; however, it is speculated that a more liberal social environment compared to 20 years ago led the patients to not comply with government orders or it might be that TB was still relatively a more common disease then but now it is considered rare and the patients may feel more socially isolated and may experience prejudice similar to that observed for other infectious diseases such as HIV or COVID-19. ^23^ Such stigma could be a psychological factor motivating patients to avoid hospitalization and should be considered in future infection control policies.

Although most infectious TB patients are persuaded to isolate and are not forcibly hospitalized, if they do not agree or leave the TB ward, law enforcement is not typically involved in their detention or pursuit in Japan. In some cases, involvement of law enforcement agencies may contribute to ensuring compliance with hospitalization in Australia, Israel, the United States, and several European countries.^29–33^ However, this measure is a sensitive issue that entails potential concerns regarding patient rights and ethical considerations. International human rights guidelines (WHO, 2017) emphasize that coercive measures should be regarded as a last resort and limited to the minimum necessary. These principles must carefully be balanced with practical needs in clinical and public health practice. Before considering coercive measures, it is preferable to strengthen alternative strategies. First, individualized counseling by public health nurses and social workers can enhance patient motivation for treatment. Second, providing economic and social support during isolation—such as assistance with living expenses, housing, or pet care—may reduce barriers to hospitalization. Third, community-based care models that utilize local support networks should be promoted. Only after these approaches are exhausted should coercive measures be contemplated as the final option.

This study represents the first investigation in Japan since 2003 regarding patients with infectious TB who resisted or refused isolation. A significant strength of this study is that questionnaires were distributed to all local health offices, with over four-fifths responding, enhancing the representativeness of our findings regarding the actual situation in Japan. However, several limitations exist. One is the reliance on questionnaire survey data, precluding verification of the reported numbers of patients with TB who resisted or refused isolation. Another is the descriptive nature of the study, which did not involve analytic epidemiology to identify factors associated with patient characteristics leading to resistance or refusal. Further research is therefore necessary to elucidate this issue.

Several recommendations are proposed to decrease the number of patients with TB who resist or refuse isolation. Firstly, the duration of hospital stay for TB patients (which averaged 44.5 days in 2022^24^) should be minimized to potentially reduce the number of patients who resist or refuse isolation. This is based on the understanding that many, though not all, patients with smear-positive TB may not transmit the disease to others or cause outbreaks.^25,26^ With the commercial availability of polymerase chain reaction-based assays for detecting rifampicin and isoniazid resistance in sputum, multidrug or rifampicin resistance can be excluded on day one of admission.^27,28^ In cases where such resistance is ruled out, the duration of hospital stay may be shortened as appropriate. Secondly, home or self-isolation might be considered for cooperative and adherent patients who comply with TB infection prevention measures and for whom continuity of treatment can be ensured. Thirdly, it is recommended that one or two facilities (likely in Tokyo and Osaka) have law enforcement officials or security guards available to enforce isolation if necessary as the last resort.
